# Cervical Cancer Screening Among Medicaid Patients During Natural Disasters and the COVID-19 Pandemic in Puerto Rico, 2016 to 2020

**DOI:** 10.1001/jamanetworkopen.2021.28806

**Published:** 2021-10-15

**Authors:** Ana Patricia Ortiz, Axel Gierbolini-Bermúdez, Jeslie M. Ramos-Cartagena, Vivian Colón-López, Kalyani Sonawane, Ashish A. Deshmukh, Karen J. Ortiz-Ortiz

**Affiliations:** 1 University of Puerto Rico Comprehensive Cancer Center, San Juan, Puerto Rico; 2 Graduate School of Public Health, Medical Sciences Campus, University of Puerto Rico, San Juan, Puerto Rico; 3 Department of University of Puerto Rico/MD Anderson Cancer Center Partnership for Excellence in Cancer Research Program, San Juan, Puerto Rico; 4 Center for Health Services Research, Department of Management, Policy, and Community Health, University of Texas Health School of Public Health, Houston

## Abstract

This cohort study examines rates of cervical cancer screening in Puerto Rico among women with Medicaid health coverage following the 2017 hurricanes, earthquakes in late 2019-2020, and the 2020 COVID-19 lockdown.

## Introduction

Puerto Rico (PR) has experienced multiple disasters in the last decade, including Hurricanes Irma and María (September 2017), a sequence of earthquakes (between December 2019 and January 2020), and the COVID-19 pandemic (starting in March 2020),^1^ all of which resulted in public health emergency declarations. In the aftermath of the hurricanes, PR residents experienced major disruptions in essential services for months, and the health care system was inoperable.^1,2^ The earthquakes led to island-wide power outages and school closings.^1^ Finally, on March 15, 2020, PR entered a COVID-19–related lockdown (executive order No. OE-2020-023) that continued until June 15, 2020 (executive order No. OE-2020-041). Quantifying cervical cancer screening disruptions is important in the context of rising cervical cancer incidence in PR.^3^ Therefore, we evaluated how the natural disasters and the pandemic factored into cervical cancer screening utilization in PR.

## Methods

This research was approved by the University of Puerto Rico Comprehensive Cancer Center institutional review board. Informed consent requirements were waived because this study used deidentified data from a government health insurance database. This study followed the Strengthening the Reporting of Observational Studies in Epidemiology (STROBE) reporting guideline for cohort studies.

We described time trends (from January 1, 2016, to July 28, 2020) in cervical cancer screening among eligible women aged 21 to 65 years using the PR Medicaid claims database. In PR, nearly half of the women aged 19 to 64 years are insured by Medicaid.^4^ We identified claims for Papanicolaou tests for women aged 21 to 29 years and Papanicolaou tests alone or with human papillomavirus cotesting for women aged 30 to 65 years^5^ using *Current Procedural Terminology* codes.^5,6^ Women with a history of cervical intraepithelial neoplasia grades 2 or 3, cervical cancer, and hysterectomy were excluded.^5,6^ Screening rates (per 100 person-months) during each calendar month were calculated, and rate ratios (RRs) were estimated to compare screening rates during each trimester in comparison with the reference period (ie, January to March 2016). Analyses were conducted using R version 4.05 software (R Project for Statistical Computing). The threshold for statistical significance was 2-sided *P* < .05 with 95% CIs.

## Results

Of a total 404 909 women, 352 520 (87.1%) were included in the cohort (mean [SD] age, 41.0 [12.7] years). Cyclic patterns of lower screening rates were observed yearly during summer and winter holiday seasons. A substantial decrease occurred in screening utilization from January 2016 (2.81 per 100 person-months) to July 2020 (0.72 per 100 person-months). Screening rates were particularly low after the hurricanes (September 2017: 1.02 per 100 person-months) and after the COVID-19–related lockdown (April 2020: 0.37 per 100 person-months) (Figure).

**Figure.**
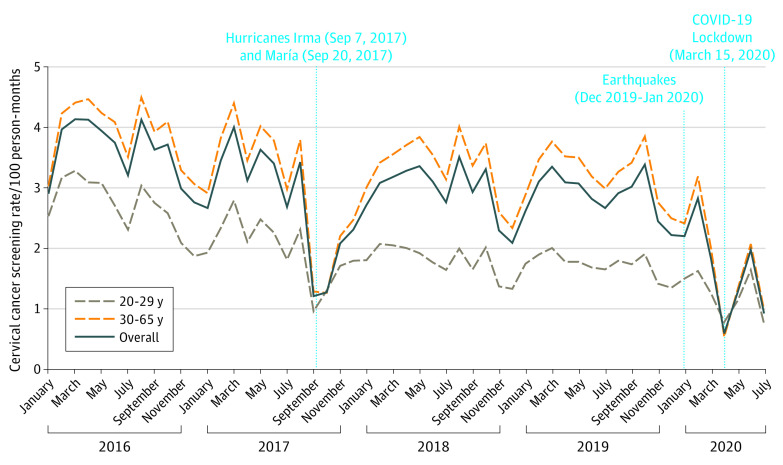
Cervical Cancer Screening Utilization Among Medicaid-Enrolled Women in Puerto Rico, January 2016 to July 2020

Screening rates among women aged 21 to 29 years dropped from 2.90 per 100 person-months (95% CI, 2.83-2.97) in January to March 2016 to 1.00 (95% CI, 0.95-1.02) during April to June 2020 (Table). Among women aged 30 to 65 years, rates for the same comparison periods decreased from 3.85 (95% CI, 3.80-3.90) to 1.10 per 100 person-months (95% CI, 1.08-1.12). Compared with January through March 2016, the greatest reductions in screening utilization were observed after the hurricanes (ages 21 to 29 years: 50% reduction; RR, 0.50; 95% CI, 0.48-0.52; ages 30 to 65 years: 52% reduction; RR, 0.48; 95% CI, 0.47-0.49) and the COVID-19 lockdown (ages 21 to 29 years: 66% reduction; RR, 0.34; 95% CI, 0.33-0.36; ages 30 to 65 years: 71% reduction; RR, 0.29; 95% CI, 0.29-0.30).

**Table.** Routine Cervical Cancer Screening Rates Among Women Aged 21 to 65 Years From the Government Health Plan of Puerto Rico, January 2016 through June 2020

Trimester	Cervical cancer screening tests			
	Age 21-29 y, Papanicolaou test		Age 30-65 y, Papanicolaou test or HPV cotesting	
	Rate (95% CI), per 100 person-months	Rate ratio (95% CI)	Rate (95% CI), per 100 person-months	Rate ratio (95% CI)
**2016**				
January-March	2.90 (2.83-2.97)	1 [Reference]	3.85 (3.80-3.90)	1 [Reference]
April-June	2.87 (2.80-2.94)	0.99 (0.95-1.02)	4.25 (4.20-4.30)	1.10 (1.08-1.12)
July-September	2.59 (2.52-2.66)	0.89 (0.86-0.93)	3.94 (3.90-3.99)	1.02 (1.01-1.04)
October-December	2.05 (1.99-2.11)	0.71 (0.68-0.73)	3.42 (3.38-3.47)	0.89 (0.87-0.91)
**2017**				
January-March	2.22 (2.16-2.29)	0.77 (0.74-0.80)	3.67 (3.62-3.71)	0.95 (0.94-0.97)
April-June	2.15 (2.09-2.22)	0.74 (0.72-0.77)	3.71 (3.66-3.75)	0.96 (0.95-0.98)
July-September^a^	1.54 (1.49-1.59)	0.53 (0.51-0.55)	2.59 (2.55-2.62)	0.67 (0.66-0.68)
October-December	1.44 (1.39-1.49)	0.50 (0.48-0.52)	1.83 (1.80-1.87)	0.48 (0.47-0.49)
**2018**				
January-March	1.83 (1.78-1.89)	0.63 (0.61-0.66)	3.26 (3.21-3.30)	0.85 (0.83-0.86)
April-June	1.75 (1.69-1.80)	0.60 (0.58-0.63)	3.65 (3.61-3.70)	0.95 (0.93-0.97)
July-September	1.60 (1.55-1.66)	0.55 (0.53-0.58)	3.44 (3.40-3.49)	0.89 (0.88-0.91)
October-December	1.41 (1.36-1.46)	0.49 (0.46-0.51)	2.80 (2.76-2.84)	0.73 (0.71-0.74)
**2019**				
January-March	1.73 (1.68-1.79)	0.60 (0.57-0.62)	3.31 (3.26-3.35)	0.86 (0.84-0.88)
April-June	1.58 (1.53-1.64)	0.55 (0.52-0.57)	3.33 (3.29-3.38)	0.87 (0.85-0.88)
July-September	1.57 (1.52-1.63)	0.54 (0.52-0.57)	3.14 (3.10-3.19)	0.82 (0.80-0.83)
October-December	1.39 (1.34-1.44)	0.48 (0.46-0.50)	2.95 (2.91-2.99)	0.77 (0.75-0.78)
**2020**				
January-March^b^	1.29 (1.24-1.34)	0.44 (0.42-0.46)	2.42 (2.38-2.46)	0.63 (0.62-0.64)
April-June^c^	1.00 (0.95-1.04)	0.34 (0.33-0.36)	1.13 (1.10-1.16)	0.29 (0.29-0.30)

^a^ Coinciding with Hurricanes Irma and María (September 7 and September 20, 2017).

^b^ Coinciding with the Puerto Rican earthquakes (started on December 28, 2020; strongest earthquake occurred on January 7, 2020, and resulted in declaration of a state of emergency) and COVID-19 related lockdown (started on March 15, 2020).

^c^ Coinciding with the COVID-19–related lockdown.

## Discussion

Cervical cancer screening rates declined among Medicaid enrollees in PR from 2016 to 2020. The greatest reductions coincided with the occurrence of the hurricanes (September 2017) and with the events that affected PR in the first quarter of 2020 (earthquakes in January and the COVID-19–related lockdown in March). Although some improvements in screening rates were observed after January 2018, these never reached the 2016 levels and plummeted with the COVID-19 pandemic. These findings are concerning because cervical cancer incidence has increased in PR in recent years (from 9.2 to 13.0 per 100 000 during 2001 to 2017).^3^ Public health efforts should focus on increasing systems of infrastructure and resilience, including the inclusion of goals and objectives that will help maintain cancer prevention and treatment services during and after disasters.^2^

This study was limited to women enrolled in Medicaid, and so these results cannot be generalized to commercial health plan enrollees. Urgent efforts are needed to recover plummeted cervical cancer screening rates and curb the rising cervical cancer burden in PR.
